# Implementation of a Rapid Post-Code Debrief Quality Improvement Project in a Community Emergency Department Setting

**DOI:** 10.51894/001c.21376

**Published:** 2021-04-13

**Authors:** Tomasz Przednowek, Camille Stacey, Katherine Baird, Robert Nolan, Jesse Kellar, William D. Corser

**Affiliations:** 1 Emergency Medicine Spectrum Health Lakeland; 2 Respiratory Therapy Spectrum Health Lakeland; 3 Emergency Medicine Saint Agnes Medical Center; 4 Statewide Campus System Michigan State University College of Osteopathic Medicine

**Keywords:** trauma, survey, wellness, emotional support, nursing, post-code debriefing, quality improvement, debriefing, code, emergency medicine

## Abstract

**CONTEXT:**

Regular debriefing has been associated with improved resource utilization and measurable improvements in team performance in crisis situations. While Emergency Department (ED) staff have often stated that they would like to be provided a formal debriefing model after “code blue” and similar events, few EDs have such protocols in place.

**METHODS:**

The study consisted of two data collection processes: (1) completion of a 7-item survey distributed pre-intervention, 6-months post-intervention, and 1-year post-intervention, and (2) completion of a Rapid Post-Code Debriefing form. Overall responses were measured on a possible 0-10 scale and individual responses were tracked. The debrief process was triggered by one of four criteria and followed a standard format using a readily available form.

**RESULTS:**

A total of 178 pre- and post-debriefing protocol implementation survey responses were collected throughout the duration of the study. Of those, 79 (44.4%) were pre-protocol response surveys. The post-protocol responses were comprised of 51 (51.5%) six month and 48 (48.5%) 12-month surveys. The average overall satisfaction with code-response performance increased significantly following the implementation of the debriefing protocol, from M=6.661, SD=2.028 to M=7.90, SD=1.359 (independent t-test = 5.069, p<0.001). There was a statistically significant decrease regarding how respondents felt emotionally supported after a code by their staff, (Pearson Chi Square 14.977, df 4, p = 0.005).

**CONCLUSION:**

During this study, implementation of a post-code debriefing resulted in increased overall satisfaction with how codes had been conducted and there was a significant change in how staff felt in regards to code team leaders and an expectation of “returning to work.” However, there a noted overall decrease in perceptions of feeling supported by other staff involved during the code. Further studies in both community and academic-based ED settings are needed to further explore these complex relationships.

## INTRODUCTION

### Background

Healthcare workers in emergency department (ED) settings are at routine risk of being exposed to potentially traumatic events and these patient care situations often have a profound impact on the staff involved. Debriefing, which allows for emotional processing and reflection upon areas for possible improvement, has been found to be one way in which to increase overall performance, reduce equipment-related problems, and improve communication and teamwork.^1,2^

However, despite being well established in the military and other high-stakes industries such as aviation, debriefing remains poorly established in the ED settings.^3,4^ Given that the benefits of post-simulation debriefings have been widely accepted in other settings, there is likely significant utility in implementing a debriefing protocol in the Emergency Department as well.^2,5,6^

### Importance

While staff have often stated that they would like to be provided a formal debriefing model after treating a cardiac arrest or other critically ill patient, few EDs have such protocols in place and there is often no formal training offered during medical education on how to debrief after a situation.^4,7–10^ When given the option, however, people usually prefer to debrief with persons facing the same stressful situation.^11^

When healthcare clinicians in these types of situations are allowed to debrief, it leads to increased empathy, normalization, and validation, which can have significant stress moderating effects.^5,12,13^ Regular debriefing has been associated with improved resource utilization and measurable improvements in team performance in crisis situations.^1,2,5,14–16^ Implementing a standardized debriefing process encourages a supportive team-based culture, improves the transition to patient care activities after the event, increases feelings of support by peers and leaders, and improves time to regroup prior to returning to work assignments.^12,17^

### Goals of This Investigation

The goals of this quality improvement project were to assess: a) the overall baseline satisfaction with how resuscitations had been performed, b) perceptions of any confusion during resuscitations, problems with lack of equipment/medications/staffing, c) level of perceived emotional support after codes, and d) reoccurrence of associated thoughts with a given code over 24 hours. The overall hypotheses were that a) baseline satisfaction with how resuscitations had been performed would improve, b) confusion during resuscitations and missing equipment/medications/staffing would decrease, c) the level of perceived emotional support after a code would improve and d) there would be a decreased reoccurrence of associated thoughts with a given code over 24 hours.

## METHODS

Before data collection, the study was determined exempt by the authors’ institutional review board in June 2018. The study consisted of a survey portion and a Rapid Post-Code Debrief form (see supplement) which were collected at two community-based Emergency Departments. The Lakeland seven-item survey was created with this goal in mind, in part based on a previous, non-externally validated survey from a previous study in an ED setting and partially created de-novo with staff input from physicians, nurses, physical therapists, and emergency technicians from the EDs participating in this study.^17^ The brief 7-item survey was distributed three times: prior to implementation of the Rapid Post-Code Debriefing, after 6 months of debriefings, and at the one-year mark post implementation.

Overall responses were measured on a possible 0-10 scale (“0” meaning “completely unsatisfied/never” and “10” meaning “completely satisfied/often”) and individual responses were tracked using an anonymous, unique ID created by each person. Each participant was also asked to identify their role on the healthcare team, and overall trends were assessed regarding responses to each of the survey items. The survey was hand distributed during morning staff meetings to each staff member working in the Emergency Department who participated in the debriefing process. These included nurses, patient care technicians, respiratory therapists, attending physicians, and resident physicians. In addition to polling during morning staff meetings, staff were also polled at various times of the day, as well as in different locations, and on different days of the week.

Survey responses, while identifiable by a unique code created by each participant, could not be linked by the researcher to any specific individual at any time since the exact code used by each person was only known by the person generating the code and they were collected in anonymous envelopes.

The debrief process was triggered by one of four criteria (see “Rapid Post-Code Debrief” form below). Forms were available at the clerk’s desk. Although the Rapid Post-Code Debrief form was most often filled out by the charge nurse or documenting nurse immediately following a qualifying event, anyone was able to initiate the process and anyone was allowed to self-exclude for any reason, such as when it was felt debriefing was a lower priority relative to other operating needs at the time (e.g., other urgent staff/patient needs). Individual names were not recorded of those in attendance, nor was this process used in any way punitively against staff who chose to participate. While there was a check box on the form regarding staff occupation, no additional demographic information (e.g., age, gender, tenure on ED staff, etc.) was collected or analyzed in this study.

Instructions on how the debrief was performed, what triggered a debriefing, and information collected on the debrief form can be seen below.

**Figure:**
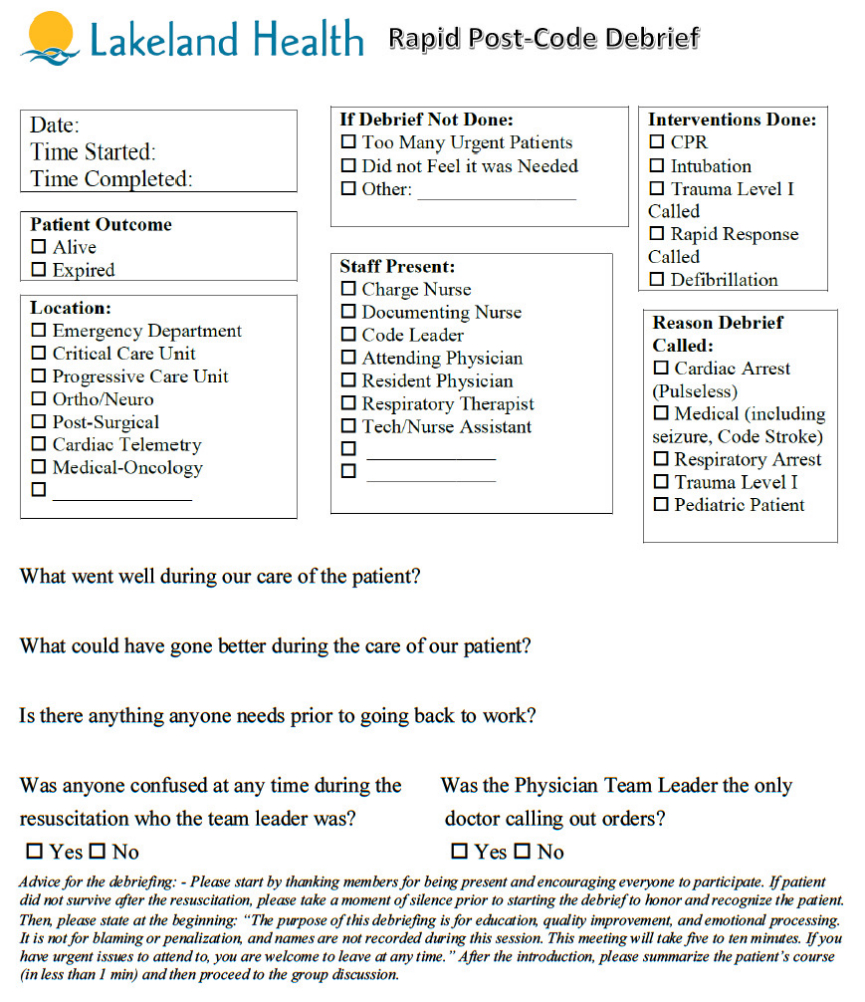


### Primary Data Analysis

After data collection was completed, the surveys were analyzed using a series of independent-sample t-tests and Chi Square crosstabulations and graphs.^18^ Due to the difficulty in tracking individual responses across the three time intervals to survey questions, all responses were categorized into thirds. Those most unhappy with lowest scores were placed into one group, there was a second group for those with a medium score, and finally those with the highest scores were in a final third group. Changes were then measured across the three groups across the study period (e.g., asking “did the bottom third of scores improve over time?”). Also, given difficulty with obtaining survey responses, many analyses (unless otherwise specifically stated) combined the six-month responses with the one-year responses as simply a “post implementation group”. A series of non-parametric stepwise multinomial regression analytic procedures were performed to examine for other potential factors influencing responses.^19^ All analytic procedures were conducted using S.P.S.S. version 25 analytic software observing a two-tailed coefficient Alpha p value of 0.05 to indicate statistical significance.^20^

## RESULTS

A total of 178 pre- and post-debriefing protocol implementation survey responses were collected throughout the duration of the study. Of those, 79 (44.4%) were pre-protocol response surveys. The post-protocol responses were comprised of 51 (51.5%) six month and 48 (48.5%) 12-month surveys. Of the 138 respondents who reported their professional role, 30 (21.7% of reported) were physicians, 56 (40.6%) were nurses and 52 (37.7%) were other types of healthcare personnel. Approximately 40 (22.5%) staff members chose not to disclose their professional roles. Every attempt was made to match participants at each of the survey intervals based on the respondent’s unique identifier, although ultimately the majority of surveys could not be matched due to either missing identifiers or the respondents not remembering their identifier.

Notably, the average overall code satisfaction pre-protocol (Mean = 6.661, SD 2.028) was significantly lower than the average of combined six and year post-protocol survey responses, or “post implementation” (Mean = 7.90, SD 1.359) (independent t-test = 5.069, p < 0.001). The average post-implementation satisfaction of respondents was 7.33 (SD 1.80) and ranged from 0 (“Not at All Satisfied”) through 10 (“Completely Satisfied”). Each survey had a total possible score calculated by adding responses to each question (with a maximum score of 60), and scores ranged from 13 through 56 on pre-implementation surveys and on the surveys at six months and one year post implementation (mean 36.89 (SD 7.30)).

Overall code satisfaction ratings increased significantly after implementation of the debriefing (Pearson Chi Square = 37.377, df 10, p < 0.001 (continuous measure), Pearson Chi Square = 16.561, df 2, p < 0.001 (categorical measure) (Table 1, Figure 1, Figure 2). Different results were observed when responses were stratified by provider type (Pearson Chi Square = 13.271, df 8, p = 0.103). Only two other survey questions showed improvement over the study period (e.g., “Number of people running a code,” and “Thoughts about returning to work”) (Table 1). Finally, there was a statistically significant decrease regarding how respondents felt emotionally supported after a code by their staff, (Pearson Chi Square 14.977, df 4, p = 0.005) (Table 1, Figure 3).

**Table:** Table 1 – Statistical Results of each Survey Item

**Survey item**	Results of Pearson Chi Square test*
Overall satisfaction	Pearson Chi Square = 37.377, df 10, **p < 0.001** (continuous measure), Pearson Chi Square = 16.561, df 2, **p < 0.001** (categorical measure)
Number of people running a code	Pearson Chi Square = 10.945. df 4, **p = 0.027**
Frequency of items missing	Pearson Chi Square = 2.648, df 4, p = 0.618
Appropriate number of staff present in the room did not change	Pearson Chi Square = 8.428, df 4, p = 0.077
Thoughts about just returning to work	Pearson Chi Square 11.351, df 4, **p = 0.023**
Recurring thoughts about the code	Pearson Chi Square 4.644, df 4, p = 0.324
Felt emotionally supported after a code by their staff	Pearson Chi Square 14.977, df 4, **p = 0.005**

* Statistically Significant Differences appear in **Bold Font**

**Figure:**
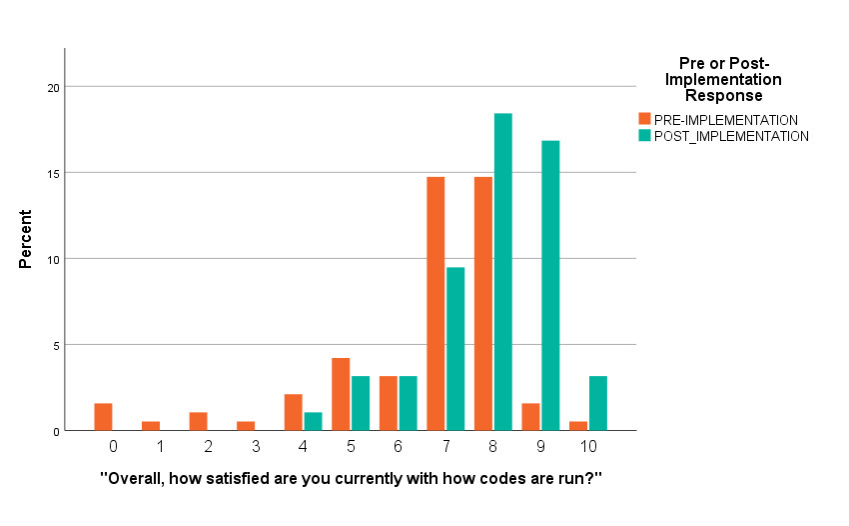
Figure 1 – Continuous Overall Satisfaction Pre-implementation vs Post-Implementation

**Figure:**
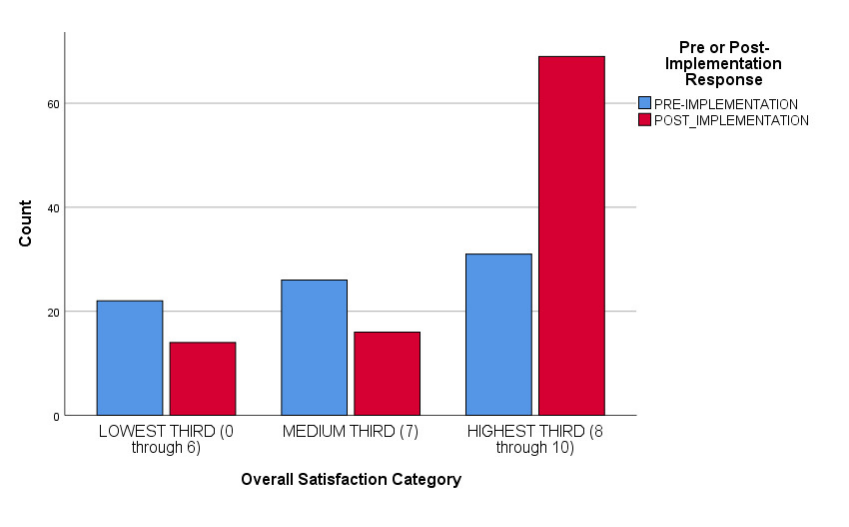
Figure 2 – Overall Satisfaction, Pre- and Post-Implementation

**Figure:**
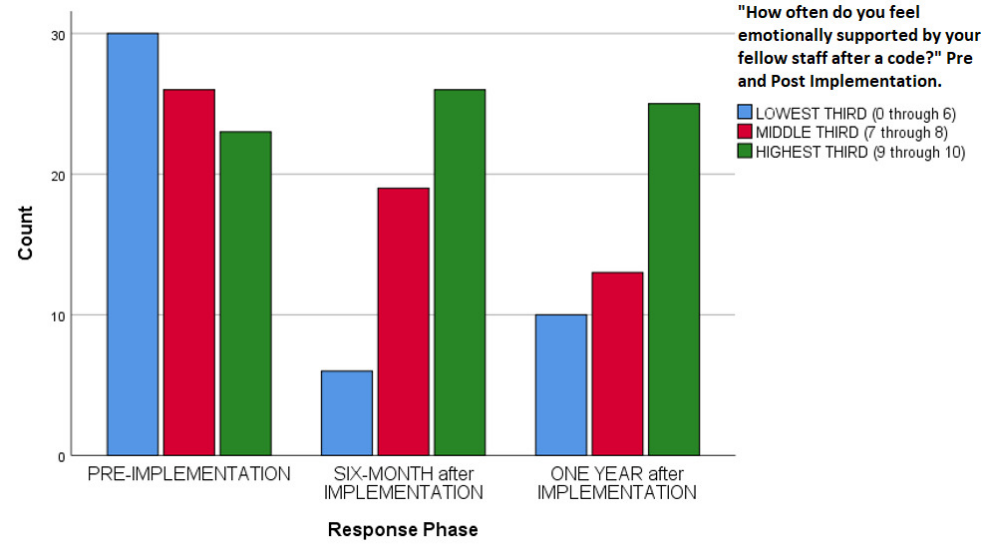
Figure 3 – Perceive Emotional Support

Finally, a series of multinomial predictive models was performed to look at the significance of each selected study measures on overall code satisfaction, and only survey time (pre-protocol vs post-protocol period), increased satisfaction significantly (Likelihood Ratio Chi Square = 16.671, df 4, p = 0.002).

## DISCUSSION

These main findings indicate that implementation of a post-code debriefing can positively impact overall satisfaction of how a code is run. In regard to the other areas assessed by the survey there were two other statistically significant changes, including a measured lower perception of emotional support after debriefing protocol implementation.

Much like in the Copeland et al. study which also implemented debriefings in the Emergency Department, the staff involved in this study also had a statistically significant improvement in overall satisfaction.^17^ Indeed, the overall response to implementation of this protocol was positive, and while it not directly measured on the survey, staff verbally indicated during morning meetings that they liked being able to debrief with persons who faced the same stressful situation, similar to what has been seen previously in the literature.^11^ The number of people running a code and thoughts about returning to work were also improved in a statistically significant manner, which is in line with previous findings such as those in the Copeland et al. study.^17^

The finding that staff felt less overall supported by other staff members after implementing the debriefing protocol is unexpected and goes against what was found in the similar previous study by Copeland et al.^17^ Perhaps this is due to the staff paying more critical attention to this aspect of a debriefing after code events and their work environment, or perhaps there may be other factors involved. Further studies may help elucidate the exact etiology behind this finding.

### Limitations

The study has several limitations. Some of these non-significant results could be attributed to lack of a sufficient-sized and/or diverse enough sample. The sample size included surveys which did not have a unique identifier which could be tracked throughout the study period, either due to the participants not including one on the form altogether or forgetting their unique ID during the three survey periods and inventing a new one each time. Results may also have been skewed by “preferred response” pressures in which some respondents may have felt pressure to indicate that the protocol helped them after code events. Additionally, these findings may not possess “external generalizability” to non-community-based ED settings.

Although every effort was made to maximize the survey response rate, it was not possible to ensure all eligible staff filled out a survey, nor could it be ensured that staff filled out surveys at all three time points. Every attempt was made to poll staff at different times of the day, in different locations, and on different days of the week. However, despite this, absolute survey response rates declined at each time interval (going from 78 (pre-implementation) responses to 51 (six-month) and then 48 (12-month) responses).

There were several debriefing-related variables which we could not control for, such as who led each debriefing, the exact timing of when the debrief was delivered, and so on. There was also no way to control when the actual events which trigger a debriefing would occur, nor was it possible to estimate what portion of critical events went without a debriefing, especially early in the project.

Cultural, gender, social, and educational background differences often play a role in how people process information and cope with acute events and can limit the amount of support a debriefing can provide.^21,22^ Furthermore, certain skills, such as high-quality CPR, may simply already be done well enough that further quality improvement processes may not have been of much benefit, thus impacting the maximum possible satisfaction scores.^23^

## CONCLUSION

During this study, implementation of a post-code debriefing resulted in increased overall satisfaction with how codes had been conducted. There was also a significant change in how staff felt in regard to code team leaders and an expectation of “returning to work.” However, there a noted overall decrease in perceptions of feeling supported by other staff involved during the code. Further studies in both community and academic-based ED settings are certainly needed to further explore these complex relationships.
